# Transcranial electric and acoustic stimulation for tinnitus: study protocol for a randomized double-blind controlled trial assessing the influence of combined transcranial random noise and acoustic stimulation on tinnitus loudness and distress

**DOI:** 10.1186/s13063-022-06253-5

**Published:** 2022-05-19

**Authors:** Mariana Lopes Martins, Tobias Kleinjung, Martin Meyer, Vithushika Raveenthiran, Zino Wellauer, Nicole Peter, Patrick Neff

**Affiliations:** 1 Department of Otorhinolaryngology - Head and Neck Surgery, University Hospital of Zurich, University of Zurich, Zurich, Switzerland; 2 Graduate Program in Cognitive Neuroscience and Behavior, Federal University of Paraiba, João Pessoa, Brazil; 3 Division of Neuropsychology, Psychological Institute, University of Zurich, Zurich, Switzerland; 4 University Research Priority Program “Dynamics of Healthy Aging”, University of Zurich, Zurich, Switzerland; 5 Centre for Cognitive Neuroscience and Department of Psychology, University of Salzburg, Salzburg, Austria; 6 Department of Psychiatry and Psychotherapy, University of Regensburg, Regensburg, Germany

**Keywords:** Tinnitus, Transcranial random noise stimulation, Acoustic stimulation, Hearing, Auditory perception, Psychoacoustics, Audiology

## Abstract

**Background:**

Tinnitus is the result of aberrant neuronal activity. As a novel treatment form, neuromodulation is used to modify neuronal activity of brain areas involved in tinnitus generation. Among the different forms of electric stimulation, transcranial random noise stimulation (tRNS) has been shown to be a promising treatment option for tinnitus. In addition, recent studies indicate that the reduction in tinnitus can be more pronounced when different modalities of stimulation techniques are combined (“bimodal stimulation”). TRNS can be used in combination with acoustic stimulation (AS), a further treatment option recognized in the literature. The aim of the proposed study is to investigate whether simultaneous tRNS and AS improve levels of tinnitus loudness and distress.

**Methods:**

The intervention consists of bilateral high-definition tRNS (HD-tRNS) over the auditory cortex combined with the application of AS which is studied in a crossover design. The visits will be performed in 26 sessions. There will be 20 treatment sessions, divided into two blocks: active and sham HD-tRNS. Within the blocks, the interventions are divided into group A: HD-tRNS and AS, and group B: HD-tRNS alone. Furthermore, in addition to the assessments directly following the intervention sessions, there will be six extra sessions performed subsequently at the end of each block, after a period of some days (follow-ups 1 and 2) and a month after the last intervention (C). Primary outcome measures are analog scales for evaluation of subjective tinnitus loudness and distress, and the audiological measurement of minimum masking level (MML). Secondary outcome measures are brain activity as measured by electroencephalography and standardized questionnaires for evaluating tinnitus distress and severity.

**Discussion:**

To the best of our knowledge, this is the first study which uses HD-tRNS combined with AS for tinnitus treatment. The crossover design permits the comparison between HD-tRNS active vs. sham and with vs. without AS. Thus, it will be possible to evaluate the efficacy of the combined approach to HD-tRNS alone. In addition, the use of different objective and subjective evaluations for tinnitus enable more reliable and valid results.

**Trial registration:**

Swiss Ethics Committee (BASEC-Nr. 2020-02027); Swiss Federal Complementary Database (kofam.ch: SNCTP000004051); and ClinicalTrials.gov (clinicaltrials.gov: NCT04551404).

## Administrative information

Note: the numbers in curly brackets in this protocol refer to SPIRIT checklist item numbers. The order of the items has been modified to group similar items (see https://www.equator-network.org/reporting-guidelines/spirit-2013-statement-defining-standard-protocol-items-for-clinical-trials/)

**Table:** 

**Title {1}**	Transcranial electric and acoustic stimulation for tinnitus: Study protocol for a randomized double-blind controlled trial assessing the influence of combined transcranial random noise and acoustic stimulation on tinnitus loudness and distress.
**Trial registration {2a and 2b}**	The study was approved in the Swiss Ethics Committee (BASEC-Nr. 2020-02027). The study is registered in the Swiss Federal Complementary Database (kofam.ch: SNCTP000004051) and in the international trial registry ClinicalTrials.gov (clinicaltrials.gov: NCT04551404). The recruitment of the subjects started on 31 March 2021.
**Protocol version {3}**	Version 3.2 of April 14, 2021.
**Funding {4}**	The project is funded by the University of Zurich and Federal Scholarship Commission for foreign students (ESKAS), through Swiss Government Excellence Scholarship (FCS) (N°. 2020.0148).
**Author details {5a}**	**Mariana Lopes Martins**. Department of Otorhinolaryngology, Head and Neck Surgery, University Hospital of Zurich, Zurich, Switzerland. Graduate Program in Cognitive Neuroscience and Behavior, Federal University of Paraiba, João Pessoa, Brazil. **Tobias Kleinjung**. Department of Otorhinolaryngology, Head and Neck Surgery, University Hospital of Zurich, University of Zurich, Zurich, Switzerland. **Martin Meyer**. Division of Neuropsychology, Psychological Institute, University of Zurich, Zurich, Switzerland; University Research Priority Program “Dynamics of Healthy Aging”, University of Zurich, Zurich, Switzerland; **Vithushika Raveenthiran.** Department of Otorhinolaryngology, Head and Neck Surgery, University Hospital of Zurich, University of Zurich, Zurich, Switzerland **Zino Wellauer.** Division of Neuropsychology, Psychological Institute, University of Zurich, Zurich, Switzerland **Nicole Peter**. Department of Otorhinolaryngology, Head and Neck Surgery, University Hospital of Zurich, University of Zurich, Zurich, Switzerland. **Patrick Neff**. Department of Psychiatry and Psychotherapy, University of Regensburg, Regensburg, Germany. Centre for Cognitive Neuroscience and Department of Psychology, University of Salzburg, Austria. University Research Priority Program “Dynamics of Healthy Aging”, University of Zurich, Zurich, Switzerland
**Name and contact information for the trial sponsor {5b}**	This is an investigator-initiated research, so the principal investigator acts as the sponsor. Tobias Kleinjung (Principal Investigator). tobias.Kleinjung@usz.ch
**Role of sponsor {5c}**	Investigator initiated the research.

## Introduction

### Background and rationale {6a}

Tinnitus is the result of aberrant neuronal activity within the auditory pathways. This activity is erroneously interpreted as sound by the auditory centers [1, 2]. It is present in 10–20% of the population, and the prevalence increases with advancing age, occurring in more than 30% of elderly people [3].

Many pathologies can result in tinnitus, the perception of which involves different areas of the nervous system, and finally results in abnormal cortical activity [1]. The structural and functional alterations are observed in the auditory cortex and non-auditory brain areas such as the cingulate cortex, dorsolateral prefrontal cortex, insula, hippocampus, caudate nucleus, and corona radiata, among others [4–7]. Tinnitus originates in the auditory system, but changes in functional connectivity and neuronal activity of non-auditory systems influence the perception, persistence, and severity of tinnitus [8].

As a treatment form, neuromodulation can be used to modify neuronal activity of brain areas involved in the perception of tinnitus [9]. There are different types of non-invasive and invasive brain stimulation [10]. Among the different forms of non-invasive transcranial electric stimulation (tES) [11–13], we differentiate between transcranial direct current stimulation (tDCS), which is the most established and widely used method applying constant direct current; transcranial alternating current stimulation (tACS) using sinusoidal current in a fixed frequency; and transcranial random noise stimulation (tRNS), which is a subform of tACS in which a range of low- and high-frequency alternating currents are generated randomly.

TRNS has previously been demonstrated to reliably increase the cortical excitability up to 1 h after a stimulation of 10 min [14, 15]. The method could also show reliable changes in cortical activity in the auditory cortex (T7 and T8) after tRNS stimulation [16]. The effect of tRNS on the subjective perception of tinnitus can be found in eight original studies which, given the novelty and putative efficacy of the method, were all recently published [17–24]. Thus, it is important to compare the differences, implications, and limitations between them. The method showed few differences between the studies, with more variations in the number of sessions. The treatment was performed using the intensity of 2 mA in seven studies [17–23] and 1.5 mA in one study [24]. All studies performed the stimulation for 20 min, either alone or in combination with other treatments [17–24]. The number of sessions ranged between one session [17, 20–22, 24], eight sessions in 4 weeks [23], and 10 consecutive daily sessions [18]. A case report conducted the stimulation with 2–3 days between sessions, although the total number of sessions was not mentioned in this report [19]. All studies used a pair of surface sponge electrodes with 35 cm^2^ placed in saline solution [17–24].

Some studies analyzed the effect of stimulation with electrodes over the bilateral temporal cortex (T3/T7 and T4/T8) [17–19, 24]; or used these positions (T3 and T4), and compared this montage with dorsolateral prefrontal cortex stimulation (F4 and FP1) [20–22]. Others compared alternative neuromodulatory techniques, for example, tDCS and tACS, in the same montage [24]; or bifrontal (F3 and F4) tDCS alone with multisite tDCS and tRNS (T3 and T4) [23].

The results were presented differently based on the objectives of each study. A study conducted by Joos et al. [17] compared different types of frequency bands used for tRNS. A decrease in tinnitus loudness and distress could be observed in the groups that received low-frequency stimulation (lf-tRNS; 0.1–100 Hz) and high-frequency stimulation (hf-tRNS; 100–640 Hz), but no substantial effect could be obtained for the group that received whole-frequency spectrum tRNS (wf-tRNS) [17]. However, it is important to note the large difference between the number of patients, lf-tRNS (*n* = 119), hf-tRNS (*n* = 19), and wf-tRNS (*n* = 16), and the absence of a control group to assess the effectiveness of the therapy [17].

Another study evaluated the tRNS alone and did not find significant efficacy of hf-tRNS, since only eight patients (27%) responded positively for the reduction of tinnitus loudness, tinnitus distress, unpleasantness, and depression [18]. A possible bias in the results was the use of repetitive transcranial magnetic stimulation (rTMS) treatment before tRNS and the absence of a sham group [18]. In a case study of tinnitus associated with red ear syndrome, substantial alleviation of pain intensity, and a prolongation of the interval between attacks was observed, although the tinnitus complaints did not change [19].

Three studies by the same authors with similar characteristics have been published [20–22]. The multisite protocol, in a sham-controlled clinical trial with lf-tRNS, observed a reduction in tinnitus loudness in the real condition, for both multisite and auditory cortex groups [20–22]. A reduction in tinnitus distress was only found in the multisite group. Multisite tRNS has been shown to be more effective in reducing tinnitus loudness and distress [21]. The other study used resting-state EEG analysis to assess lf-tRNS stimulations effects. In the multisite tRNS group (10 min tRNS in auditory cortex – T3 and T4 + 10 min of dorsolateral prefrontal cortex – F4 and FP1), an increase in power was identified in the alpha-1 band at the auditory and prefrontal cortex accompanied by a decrease in power in the delta and beta-2 bands in the prefrontal cortex, anterior cingulate cortex, and the parahippocampus. Furthermore, decreased alpha connectivity between the right prefrontal cortex and the left auditory cortex was observed in the same group. However, the study did not test a group with dorsolateral prefrontal cortex (DLPFC) stimulation alone [20]. In the comparison between two different groups receiving multisite tRNS, comparing 1 session (*n* = 17) vs. eight sessions over 4 weeks (*n* = 12), a reduction in tinnitus loudness and distress occurred in both groups, but with a higher suppression in the multiple-session group [22].

To et al. [23] compared different types of TES in three groups: one group received bifrontal tDCS, the second group received multisite treatment of bifrontal tDCS before bilateral auditory cortex tRNS, and the last group was the waiting list. A larger suppression effect was shown for the multisite group (loudness 21.26%, distress 25.90%) when compared with the bifrontal tDCS group (loudness 14.20%, distress 13.03%) and the waiting list group (no effect). The study is limited in the association of tDCS and tRNS, which makes it impossible to analyze the techniques separately [23]. When comparing three different techniques (tDCS, tACS, and tRNS), the tRNS induced the larger transient suppressive effect on tinnitus loudness and distress, and no significant effect was obtained for tDCS and tACS. Thus, results indicate that tRNS may be the most effective single session method for the transient suppression of tinnitus [24].

Acoustic masking is an established method for treatment or alleviation of tinnitus. A competitive acoustic signal is used to modify the perception of tinnitus at a higher cortical level [1]. Acoustic stimulation (AS) in the form of maskers [25], but more elaborated forms for acoustic neuromodulation [26–28], are commonly used. While acoustic neuromodulation and masking requires a relatively long time to induce any effect and is still the subject of critical discussion, residual inhibition (RI) is a well-established principle which can be induced in most tinnitus sufferers after short acoustic stimulation (AS) with white noise (WN) or other sounds. The gradual suppression of tinnitus during a period of RI has been proposed to reflect the resumption of synchronous activity in frequency regions of the auditory cortex [29].

The combination of two or more kinds of treatments, known as bimodal or multimodal stimulation, can be effective for the induction of beneficial neuroplastic effects [9]. A pilot study conducted by Shekhawat et al. in 2015 [30] tested the effects of simultaneous electric and acoustic stimulation.

An improvement in the level of tinnitus perception using tDCS and bilateral broadband noise simultaneously was found in comparison to tDCS or sham alone. Further similar approaches have been published in recent years, namely pilot studies [31, 32], study protocols [30, 33], and a single experimental study [34]. Their results indicate a superior efficacy of electric stimulation combined with acoustic approaches, which highlights the need to conduct large-scale controlled studies to verify these findings.

The approach we propose here, similar to the bimodal approaches above, aims to couple the effects of high-definition tRNS (HD-tRNS) and AS for better temporary tinnitus suppression and a possible reversal of the maladaptive neuroplasticity related to tinnitus. We aim to target the (bilateral) auditory cortex with tRNS, as in former studies [17, 18, 20, 24, 35], and combine it with AS. This specific combination is novel and is based on cortical excitability following tRNS [36] which may lead to more pronounced and sustained effects.

Due to the novelty and complexity of the study and the data to be collected, the absence of any intact local patient or self-help community, and finally the clearly defined outcomes (i.e., tinnitus loudness and distress reductions), public or patient stakeholders could not be involved in the development of the study design.

The number of treatment sessions per condition is in line with well-established general and tinnitus-specific numbers of sessions [18]. Generally, TES can be considered a promising treatment for tinnitus and is practiced in centers around the world, with tDCS being the most widely used method. Research into new protocols and the refinement of established methods is still ongoing, so no specific protocols are recommended as yet [37, 38]. Furthermore, it is important to note that a published guideline for tinnitus treatments reported no evidence for the efficacy of electric stimulation on tinnitus [39].

### Objectives {7}

The proposed project aims to shed more light on the possible effects of simultaneous electrical and acoustic stimulation in tinnitus treatment. Since tRNS has been shown to be the most effective tES method [24], and maskers are a well-established, standard acoustic treatment [25], we want to investigate the potential suppressive effects of these methods combined.

#### Primary objective

The primary objective is to investigate whether simultaneous HD-tRNS and AS improve the levels of subjective tinnitus perception and distress in a clinical population of tinnitus sufferers.

#### Secondary objectives

The changes in neuronal activity patterns after stimulation are to be investigated with regard to the basic mechanisms of tinnitus and treatment effects. Resting state and auditory event-related potential EEG [40] will be recorded for that purpose, as well as to be able to study the putative relationship between brain activation patterns and the subjective tinnitus perception (i.e., tinnitus loudness and tinnitus-related distress) as evaluated by standardized questionnaires and rating scales.

### Trial design {8}

The study is a double-blind, pseudo-randomized (stratified randomization), sham-controlled, crossover within- and between-subject design with two study arms (i.e., testing 2 protocols vs. sham).

**Fig. 1:**
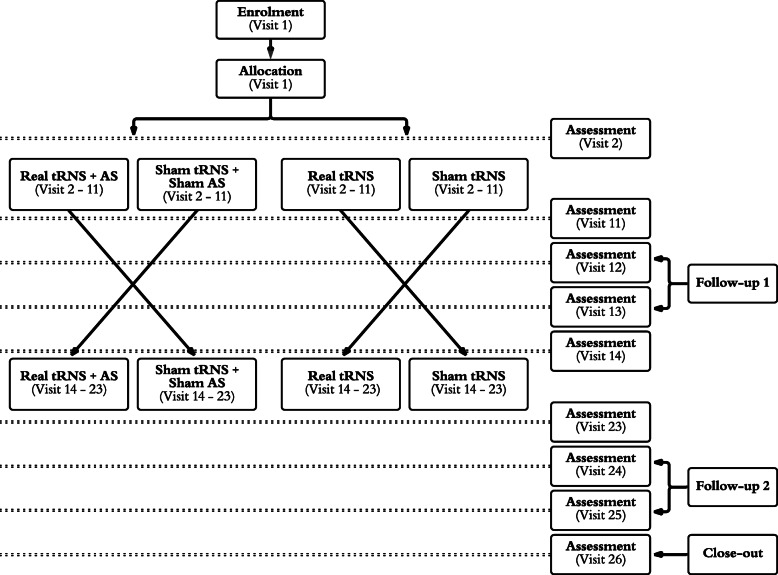
Flow diagram for crossover of randomized trial. Adapted of CONSORT, 2010

Overall, participants will undergo 20 stimulation visits and 6 additional assessment visits (Fig. 1).

The present protocol follows the Standard Protocol Items Recommendations for Interventional Trials (SPIRIT) guidelines and fulfills the SPIRIT checklist (https://www.equator-network.org/reporting-guidelines/spirit-2013-statement-defining-standard-protocol-itemsfor-clinical-trials/; Accessed 10 June 2021).

## Methods

### Study setting {9}

Single-center study of the University Hospital Zurich (USZ) in cooperation with the University of Zurich (UZH).

The study has been approved by the local ethics committee with registration number BASEC-Nr. 2020-02027. Furthermore, the study is registered in the Swiss Federal Complementary Database (kofam.ch: SNCTP000004051) and in the international trial registry ClinicalTrials.gov (clinicaltrials.gov: NCT04551404).

### Eligibility criteria {10}

#### Inclusion criteria

Participants fulfilling all of the following inclusion criteria may be enrolled in the study:

- Male and female participants between 18 and 75 years of age
- Persistent chronic tinnitus with duration of more than 3 months [41]
- Signed Informed Consent after being informed about the study
- Fluent in German
- Tinnitus with a Tinnitus Handicap Inventory (THI) Grade 2 to 4 (18–76 points)
- Willing and being able to attend the study visits

#### Exclusion criteria

The presence of any one of the following exclusion criteria will lead to exclusion of the participant from the study:

- Current neurological or psychiatric disorders
- Hyperacusis
- Regular intake of medication influencing the central nervous system (e.g., neuroleptics, hypnotics, sedatives, and anti-epileptics)
- Implanted pacemaker
- Surgical implants in the head region, such as cochlea implants
- Asymmetrical hearing (> 20 dB difference between sides), pantonal hearing loss (> 40 dB in any measured frequency up to 2 kHz)
- Women who are pregnant or breastfeeding
- Intention to become pregnant during the course of the study
- Known or suspected non-compliance, drug or alcohol abuse
- Participation in another study with an investigational drug within the 30 days preceding or during the present study
- Enrolment of the investigator, his/her family members, employees and other dependent persons (recommended exclusion criterion by the Swiss Ethics Committee)

#### Dropout criteria

The dropout criteria are participants who discontinue the study without justification.

### Who will take informed consent? {26a}

All participants must agree to contact with the informed consent form before they are submitted to the study procedure. Before the first session, the study personnel explains the aim of the study, the procedures, the duration of sessions, and potential risks and benefits. The participant is also informed that participation is voluntary and that a withdrawal from the study will not affect the treatment or have any other consequences for the participant. Furthermore, withdrawal from the study is possible any time without the obligation to indicate any reason for this decision.

The information is provided in writing and explained verbally by the researcher. The participant should read and understand before dating and signing the document. After approval, a copy of the signed document is given to the participant.

### Additional consent provisions for collection and use of participant data and biological specimens {26b}

This is not applicable.

## Interventions

### Explanation for the choice of comparators {6b}

The study intervention consists of a bilateral HD-tRNS application over temporal regions, parallel to the application of AS in one study arm. Stimulus intensity will be below individual sensation threshold, with a max. of 2 mA. AS will never surpass 85 dB SPL at the ears.

#### Study intervention(s) group A

- HD-tRNS bilateral temporal regions combined with AS for 20 min
- Sham HD-tRNS bilateral temporal regions combined with Sham AS for 20 min

#### Study intervention(s) group B = control intervention

- HD-tRNS bilateral temporal regions for 20 min
- Sham HD-tRNS bilateral temporal regions for 20 min

Sham conditions are planned for both study arms to test the interventions with respect to placebo effects. The standardized sham HD-tRNS protocol of the DC-Stimulator will ramp up to 2 mA current for 30 s before ramping down to 0 mA to elicit the sensation of a faint tingling on the scalp to imitate the sensation of active tES. The AS sham condition is purposely constructed to present very low sound energy. The tES sham protocol is standard and thus best practice in basic and clinical research whereas the Sham AS is novel in its conception and use (i.e., no specific references available).

### Intervention description {11a}

The study is to be performed in 26 sessions. There are 20 treatment sessions, divided into two blocks, and 6 extra sessions for assessments (Fig. 1, 4).

In order to recruit candidates for the study, those interested in participating are screened by telephone interview. The purpose of this is to assess whether the participant fits the general inclusion/exclusion criteria and to assess their motivation to participate in such a trial. If they are interested in participating in the study, signing the informed consent and the first visit will be performed by one of two dedicated physicians of the Department of Otorhinolaryngology - Head and Neck Surgery at the USZ. Inclusion and exclusion criteria are checked during the second visit. If these are not met, the subject will be excluded from the study. If the participant can be included in the study, their anonymous data is referred to the biometrician for random assignment to study intervention group A or B (Fig. 1).

Treatment period 1 starts with the second session (the first visit of the treatment), which is divided into three stages. In the first stage, the primary and secondary outcome measures are collected on a secure online platform (for more details about the questionnaires, see section “Outcomes {12}” and Fig. 4). In the second stage, the audiological examination and EEG are performed. In the third stage, the first run of treatment is performed. Participants receive the same treatment in sessions 3–10, with intervals of 2–3 days between sessions. After each session, the participant answers a safety questionnaire in order to identify possible side effects of the TES. The 11th session is the final day of treatment in the first block, where the questionnaires of primary outcome measures are applied.

Follow-up 1 starts with session 12, in which the questionnaires of primary outcome measures are repeated in addition to the measurement of minimum masking level (MML), secondary outcome measures (EEG), and questionnaires of other outcomes measures are collected. In session 13, the questionnaires of primary outcome measures are used. This ends the first block of follow-up (Fig. 4).

The second block of the study consists of Treatment Period 2 and Follow-up 2. This block has the same characteristics in the procedure and performance time as the previous sessions (from 2 to 13), the difference is the type of stimulation of HD-tRNS, which contrasts with the one previously carried out (Active/Sham). In session 14, the second period of treatment starts, and the same assessments are made as in session 2. Sessions 15–22 are treatment sessions. In session 23, the same assessments are made as in session 11. Follow-up 2 occurs in sessions 24 and 25, with the same characteristics as follow-up 1 (Fig. 4).

The Close-out is performed in session 26, 7 days after Follow-up 2 and approximately 1 month after Treatment 2 (Fig. 4). In this last session, the primary, secondary, and other outcome measures are performed.

The intervention and evaluation procedures, as well as the explanations regarding the choice of paradigms, can be found in detail below:

#### High-definition transcranial random noise stimulation (HD-tRNS)

The medical device (MD) used for HD-tRNS in this study is a Soterix Medical Multichannel Neuromodulation Stimulator (MxN-33 system, model-no. 3200C; Soterix Medical Inc, New York, USA).

Stimulation is directed to the primary auditory cortex bilaterally [42], over Heschl’s gyrus. Localization is supported by Rademacher et al. [43] in the standard MNI coordinate system.

**Fig. 2:**
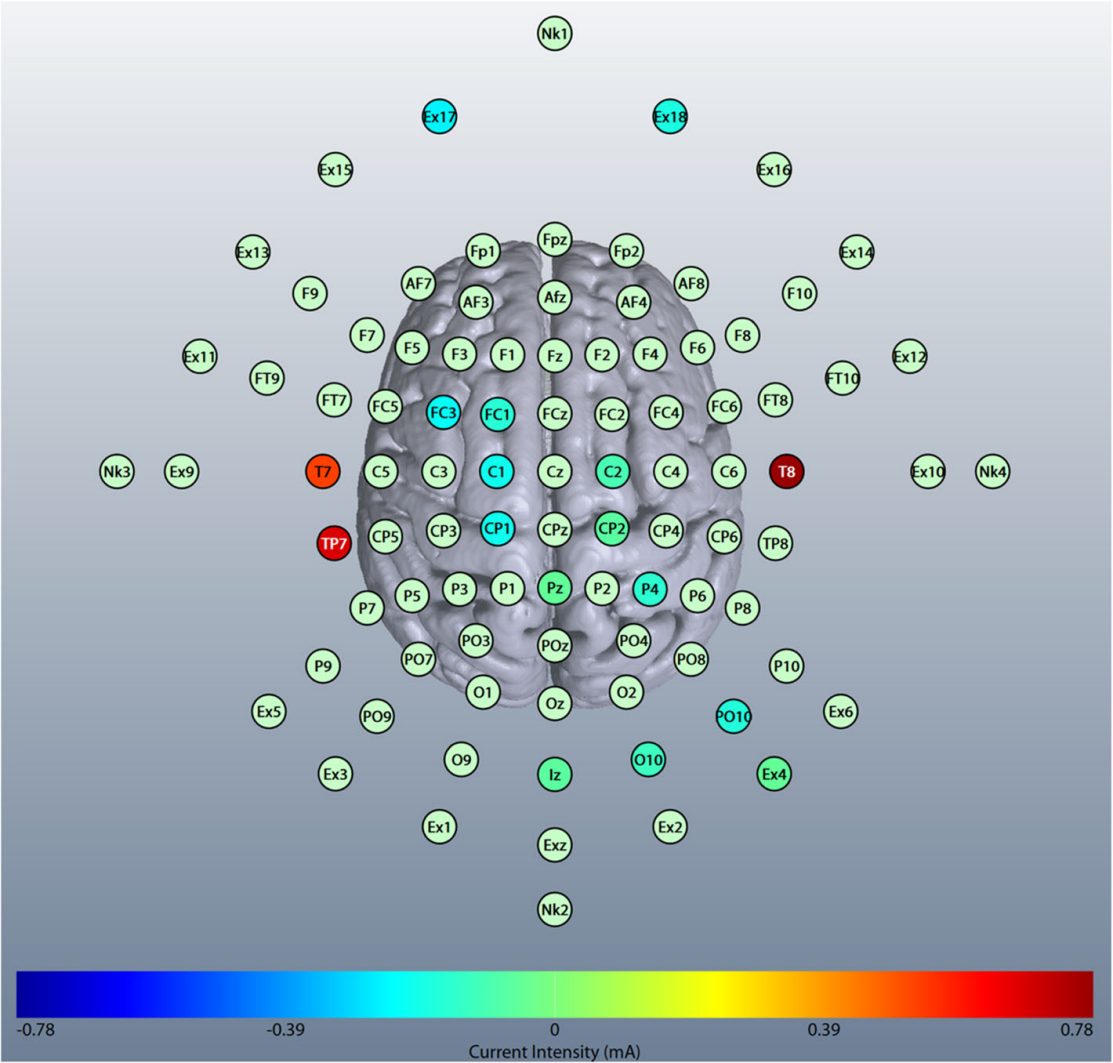
Sites of stimulation in HD-tRNS. Adapted from the manual of the system 10/20 [44, 45]. Created from the HD-Targets Software of Soterix Medical Multichannel Neuromodulation Stimulator (MxN-33 system; model-no. 3200C)

**Fig. 3:**
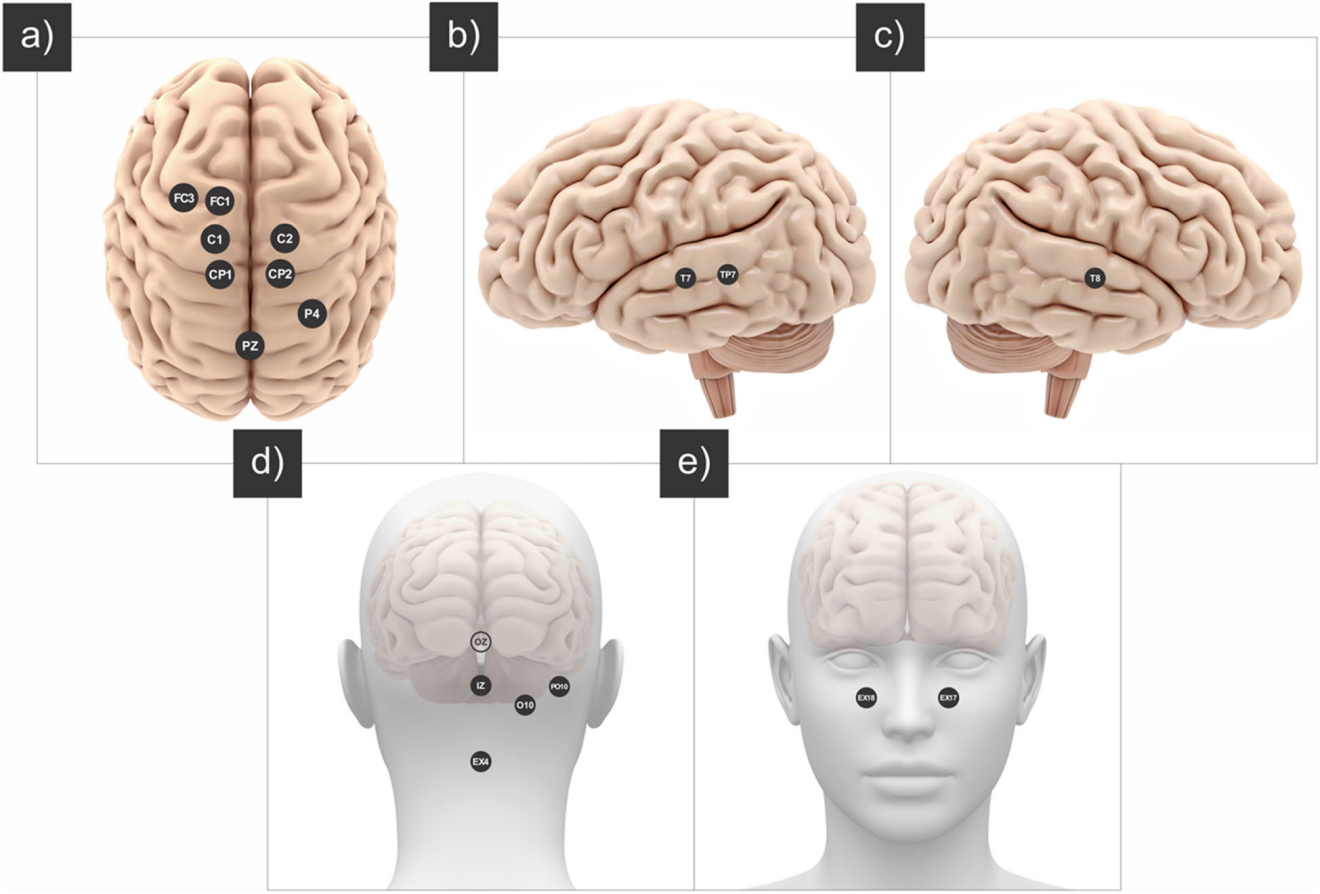
Positioning of the electrodes in HD-tRNS. Based on the 10/20 system for EEG

The Talairach coordinates of the location are reflected in the center of Heschl’s gyrus, with the targeted MNI voxels *x* = − 42, *y* = − 21, *z* = + 7 for the left and *x* = + 46, *y* = − 13, *z* = + 8 for the right side (Figs. 2 and 3). Targeting was optimized with respect to the mentioned target voxel and upper limit of total current (i.e., 2 mA) with Soterix Software MxN HD resulting in a target current of 0.095 V/m in the left and 0.072 V/m in the right target of interest.

lf-tRNS frequency range (0.1–100 Hz) will be used with seventeen channels corresponding to the positioning of the electrodes in the 10/20 system [44, 45] and their respective amplitudes TP7 (0.69 mA), T8 (0.78 mA), T7 (0.53 mA), PZ (0.05 mA), PO10 (0.17 mA), P4 (0.15 mA), IZ (0.06 mA), FC3 (0.22 mA), FC1 (0.16 mA), EX18 (0.18 mA), EX17 (0.25 mA), EX4 (0.05 mA), CP2 (0.07 mA), CP1 (0.20 mA), C2 (0.10 mA), C1 (0.21 mA), and O10 (0.12 mA) (Figs. 2 and 3). The stimulation will be applied for 20 min (totaling 21 min with ramp up and ramp down).

#### Acoustic stimulation (AS)

For acoustic stimulation, WN with an intensity of MML + 10 dB is used. The AS is also applied for 20 min and will never surpass 85 dB SPL at the ears.

For Sham AS, deep pure tones at SL + 10 dB are used, varying randomly between [1] 100 and 200 Hz [2], 1.33 and 4 Hz presentation rate [3], randomly varying in intensity ± 10% around the SL + 10 dB level, and [4] randomly varying linear decay envelope between 250 and 750 ms. The AS sham condition is purposely constructed not to cover the tinnitus pitch range and to present very low sound energy, so as not to be able to generate tinnitus suppression. The randomization of frequency, temporal, and intensity parameters ensures the absence of any regularities (including musical properties).

The AS is performed using Etymotic ER-2 research-grade, air conduction in-ear headphones (Etymotic Research Ins., USA), as with these it is possible to get quasi-linear response in frequencies up to 14 kHz compared to other air tube headphones. The intention behind the use of these headphones for AS and the auditory paradigm is to optimize the transmission of the acoustic signals and the absence of ferromagnetic metal in respect to not interfere the EEG measurements or tES being provided at the same time.

#### Electroencephalogram (EEG)—resting state

EEG is recorded with a Biosemi ActiveTwo EEG system (MarkII System, Biosemi, Amsterdam, the Netherlands) with 128 electrodes in a sound-proof and electrically shielded room. Impedances are kept below 20 kOhm. Electrodes are located according to the international 10/20 system [44, 45].

The participant is positioned in a relaxed posture and position, in front of a computer screen showing a fixation cross at eye level. The participant is told to relax and do nothing. There now follow 10 recordings of 60 s each, for a total of 10 min, with participant instructed to open and close their eyes by a soft female voice (https://tinnet.tinnitusresearch.net/images/pdf/WG3/Standardisation_Report_V5.pdf). A fixation cross is presented in open eye phases to fixate the gaze and counteract eye movements.

#### Electroencephalogram (EEG)—event-related potentials (ERP)

The Auditory Multifeature Paradigm, generated by transcriptions created in MATLAB Software (Mathworks, Natick, MA, USA) [46], is used.

Stimulus creation and presentation are performed with the Psychophysics Toolbox (PTB [47–49];) and a class-based library derivative o-PTB [50]. The research-grade, Etymotic ER-2 air conduction headphones are driven by Babyface Pro FS sound card (RME Fireface UCX; Audio AG, Haimhausen, Germany).

For each subject, two stimuli with different acoustics frequencies are presented, one at the tinnitus frequency, assessed by measurement of tinnitus pitch matching (see below), and one at 500 Hz as the standard frequency [51].

In contrast to the study of Mahmoudian et al. [52], we used pure tones (PT) instead of harmonic tones (HT) (3 partials) to ensure that the stimuli are fully heard at high frequencies, which would not be possible with HT with octaved partials (e.g. 8000, 16000, 24000).

Standard stimuli are composed of 1 sinusoidal 500 Hz tone with a total duration of 75 ms including 5 ms rise and 5 ms fall time (cosine ramp), as in the study by Asadpour et al. [53]. The same stimulus parameter set is used for the pitch-matched tinnitus frequency.

The parameters follow the auditory multi-feature paradigm by Näätänen et al. [54] who used 8 different types of deviant stimuli clustered in 5 different classes. Additionally, we added a sixth deviant class, namely PT in pink noise (PN), as seen in Pakarinen et al. [55]. The deviant tones differed from the standards in (1) Frequency—half of the frequency deviants were 10% higher, half 10% lower than the standard, (2) Intensity—half of the intensity deviants were + 10 dB and half − 10 dB compared to the standard, (3) Location—half of the deviants were mapped to the right channel and half to the left channel, (4) Duration—the duration deviant was 25 ms in length, (5) Silent gap in the middle of the tone—the gap deviant cuts out 7 ms from the middle of the standard stimulus, leaving a gap, and (6) Noise-level deviant—14 dB of PN added to the standard tone [55].

All stimuli are presented diotic at 40 dB above individual sensation Level (SL) for the two frequencies. The multi-feature paradigm consists out of 4410 tones in total and can be divided in 6 sequences (3 per frequency) which are presented in a pseudo-randomized fashion, starting with either 500 Hz or the tinnitus frequency sequence. Each sequence starts with 15 standards, followed by 60 blocks of 12 tones. The blocks include each deviant class once and every second tone is a standard. Between the blocks, the same deviant class never follows each other. In total, 735 tones are presented per sequence, so 2205 tones per frequency leading to a set of 1080 data points of deviants. Every class of deviants has the same probability of occurrence. The stimuli are presented with a stimulus onset asynchrony (SOA) of 500 ms. This means that every 500 ms from onset a tone was presented regardless of its length, keeping a 120 bpm rhythm. Every sequence lasts 367.5 s. With 10 s of break between each block, the full duration of the entire paradigm is 2245 s (~ 37 min).

## Audiometry

Audiometry comprises pure tone audiometry (PTA), loudness discomfort level (LDL), isometric forward masking contour (IFMC), temporal masking curve (TMC), and sensation level (SL) of the ERP stimuli. The tests were performed using MATLAB Software (Mathworks, Natick, MA) [46], a modified multiThreshold toolbox (University of Essex, UK), Babyface Pro FS sound card (RME Fireface UCX; Audio AG, Haimhausen, Germany), and headphones model AKG K271 MK II (AKG Acoustics, Northridge, USA). The SL is performed using Etymotic ER-2 research-grade, air conduction in-ear headphones (Etymotic Research Ins., USA) with 13 mm ear tips.

### Pure tone audiometry (PTA)

Audiometry 125 to 14,000 Hz is performed using the single-interval adaptive procedure (yes/no method) implemented in multiThreshold [56, 57]. The starting value for the target tone is at 30 dB SPL, with target duration of 250 ms.

The procedure consists of changing the level of the stimulus from trial to trial using a one-down, one-up adaptive procedure [57]. The start level is randomly located in a range 10 dB to the start value. After the first no-response, the stimulus level is set to the mid-point between the previous two levels. The procedure then continues with a reduced step size of 2 dB.

Catch trials serve to check that the participant understands the instructions. The rate of catch trials starts at 0.2. At the beginning of a run, the catch trial rate is 0.2 (20%). If the participant is caught out, the rate is increased on the next run in steps of 0.1. If the subject is not caught out on a run, the rate is reduced progressively to the minimum rate.

### Loudness discomfort level (LDL)

The LDL is used to measure the loudness at which external sounds become uncomfortable [58] and is a valid clinical measure for characterizing the “threshold of discomfort.” The participant will evaluate according to their personal perception whether they find the loudness of the sound pleasant, loud, or unpleasant. As soon as it is selected as being unpleasant, the discomfort threshold is reached, which completes a run.

The measurement is performed in each ear separately, with a PT of 500 ms at frequencies of 500, 1000, 2000, and 4000 Hz [59]. The starting level is at 75 dB and is then raised by 3 dB with each trial until the subject declares the tone is uncomfortable. The trial is then stopped, and the next trial initiated.

### Isometric forward masking contour (IFMC)

The forward masking is a technique for inferring human cochlear compression [60], and the technique is also used to detect the occurrence of subjective tinnitus as a result of cochlear damage [61, 62].

The supra-threshold measure of frequency selectivity is assessed using a forward masking procedure. A forward masking task identifies the maximum loudness level of masking tone at which the probe tone presented immediately after can still be heard, and at which the target tones are preceded by masker tones. The masker level is varied adaptively between trials to identify the masked threshold, in order to produce an isometric forward masking contour (IFMC) [63].

The initial target level is set to 35 dB for both frequencies. The parameters of the paradigm consist of target tones of 16 ms at the two frequency conditions multiplied with the factors 0.5, 0.7, 0.9, 1, 1.1, 1.3, and 1.6 preceded by a masker tone of 108 ms, with a silent gap of 30 ms in between. Thus, the participant will hear clicks (probe tones) and beeps (masker tones). The participants should report whether or not they heard the probe tone during the measurement [63, 64].

### Temporal masking curve (TMC)

The test is used to estimate auditory compression using a forward masking paradigm. The gap between the masker and the probe is varied between measurements while the frequency is held constant. A fixed-level target follows a masker tone and the audibility of the target is manipulated by changing the level of the preceding masker. Then, the masked thresholds generate a temporal masking curve (TMC) [63, 64].

For the TMC, the starting level of 50 dB is used at the frequencies of 500 Hz and the tinnitus frequency. The target stimulus is 20 ms, preceded by a 108-ms masker. The gap between the masker and the target varies between runs over a range of 5, 10, 20, and 40 ms.

### Sensation level (SL)

The SL is performed in a diotic form, with stimuli of 75 ms. The starting level is 30 dB and the stimuli are related to target frequency of 500 Hz and the individual tinnitus frequency, determined previously through matching. The procedure is similar to that used to determine the hearing threshold (PTA), but only the two specific frequencies are used.

## Tinnitometry

Tinnitometry comprises a battery of tests as pitch matching (PM), minimal masking level (MML), and residual inhibition (RI) measurement [65].

The audio system used for the tinnitometry is a custom software tool made in MAX 8 (Cycling’74, USA), using Babyface Pro FS sound card (RME Fireface UCX; Audio AG, Haimhausen, Germany), and headphones model AKG K271 MK II (AKG Acoustics, Northridge, USA), with a hardware controller Novation Nocturn USB Midi (Novation, UK) to manipulate parameters.

### Pitch matching (PM)

In the PM procedure, the participant identifies an external sound which is most similar to the subjective perception of the tinnitus. The procedure is performed using the method of adjustment [29, 66] with the same modifications described in [67].

The tinnitus frequency is identified in the ear contralateral to the tinnitus in unilateral cases, or in the better-hearing ear in cases of bilateral tinnitus [68]. The starting frequency is chosen from the audiogram and corresponds to the frequency with the highest hearing loss in each ear. The participant is then instructed to adjust the frequency to the pitch of their tinnitus, which ranges from 30 to 18,000 Hz. After identifying the frequency, the loudness is adjusted. The participant manipulates the knob corresponding to the sound intensity until they judge that the intensity emitted by the equipment is equal to that of their tinnitus.

The next step is to perform the octave confusion test. The selected frequency is compared to a frequency one octave higher and one octave lower, with the aim of confirming the frequency chosen.

To ensure reliability of the pitch matching, the test is repeated twice, and the frequency, which is matching the subjective tinnitus perception better, is chosen for further procedures.

### Minimum masking level (MML)

The MML measures the level of sound needed to cover the perception of tinnitus. It is adjusted to the lowest level of noise (white noise—WN) until tinnitus is thoroughly masked, in a diotic presentation [65, 69].

### Residual inhibition (RI)

RI is a temporary reduction of tinnitus following the cessation of masking sounds. RI measurement is performed using WN sound presented at MML + 10 dB level [30, 66]. The procedure is performed diotically.

After stimulation offset, the participants are asked to rate the change in tinnitus intensity right after the offset on a scale of − 5 (totally disappeared), 0 (same level), or + 2 (much louder).

### Criteria for discontinuing or modifying allocated interventions {11b}

Given the general low risk associated with the study procedures, no realistic scenario of withdrawal or discontinuation can be drawn for this study. Although risk is not commonly reported with the current therapy, participants will be monitored in case they experience any symptoms. The probability exists for some minor side effects exists, as reported in the literature, such as nausea, tingling, headache, tiredness or irritating sensation, difficulty in concentration, redness, or pain [18].

In any case, participants may terminate their participation in the study at any time and without any justification. They will be registered as dropouts and will not be taken into account in the analysis.

All participants will be informed in writing on the information sheet and on the informed consent form, and verbally in the interview prior to enlisting in the study, that they may terminate their participation in the study at any time and without justification. After withdrawal, the participants will be contacted for a debriefing interview. Dropouts will be replaced in order to achieve the intended sample size whenever possible. Replacement will be performed along the established selection and allocation criteria.

Compliance is usually high, as has been observed in former studies of our group [70, 71], and other groups in the field of tinnitus research. This is true even in the absence of monetary incentives and possible detrimental effects on the condition. Therefore, no specific measures are considered to increase compliance.

### Strategies to improve adherence to interventions {11c}

Participants will be contacted by phone and email, then appointments will be scheduled in advance according to the availability of each participant. In order to improve adherence, appointments will be available in three shifts, from Monday to Saturday.

The questionnaires will be collected on an easy-to-navigate online platform before each session at which the participants are present and in an online form in sessions 13 and 25. Additionally, after each therapy session, a safety questionnaire will be applied in order to identify any side effects resulting from the stimulation.

In case of non-compliance, such as absence in sessions, the participants will be contacted via telephone or email, and asked about continuation or early termination of the study.

### Relevant concomitant care permitted or prohibited during the trial {11d}

During the recruitment and before each treatment session, the participants are asked not to participate in any concomitant interventions during the study.

All urgent interventions or treatments are recorded in the research documentation.

### Provisions for post-trial care {30}

Provisions for ancillary and post-trial care are not relevant to the study.

### Outcomes {12}

#### Primary outcome

There are three primary outcome measures in the study. Two of them assessed by means of behavioral self-report, visual analog scale (VAS) ratings of tinnitus for loudness and distress [72], and the third is performed by measuring the MML.

#### Secondary outcome

The secondary outcome is resting state and event-related EEG potentials [40] recorded for that purpose.

#### Other outcome of interest

**Fig. 4:**
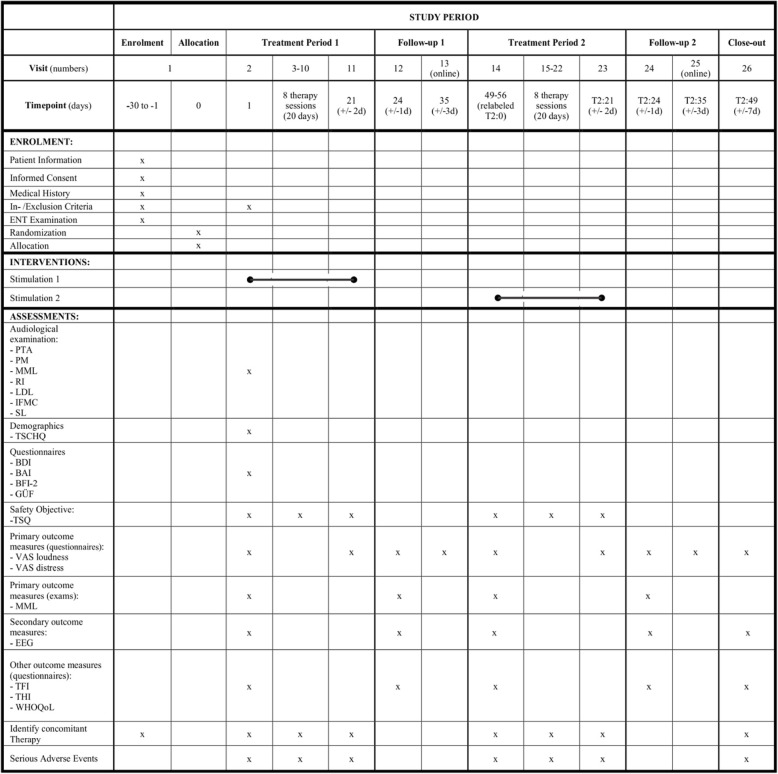
Schedule of enrolment, interventions, and assessments. Adapted of SPIRIT, 2013

The other outcomes will be used in order to describe and characterize the study sample, control variables and correlational analyses. These will be assessed only on the second visit, before the first treatment (Fig. 4).

To complete primary and secondary outcomes, the standardized Tinnitus Case History Questionnaire [72], physical health, psychological health [73–75, 77, 79, 80], and personality questionnaires [76, 81], as well as standardized tinnitus and hyperacusis questionnaires [72, 82–86] are assessed. Audiometry and tinnitometry complement this set of outcomes.

##### Tinnitus and hyperacusis

- Tinnitus Sample Case History Questionnaire (TSCHQ) [72]
- Tinnitus Functional Index (TFI) [78, 83]
- Tinnitus Handicap Inventory (THI) [84, 85]
- Geräusch-Überempfindlichkeits-Fragebogen (GÜF) [86]

##### Audiometry/tinnitometry

- Pure tone audiometry (PTA)
- Pitch matching (PM)
- Minimum masking level (MML)
- Isometric forward masking contour (IFMC)
- Temporal masking curve (TMC)
- Sensation level (SL)
- Loudness discomfort level (LDL)
- Residual inhibition (RI)

##### Physical and psychological health

- Beck-Angst-Inventar (BAI) [73, 77]
- Beck-Depressions-Inventar (BDI) [74, 79]
- World Health Organization Quality of Life (WHOQoL) [75, 80]

##### Personality

- Big Five Inventory (BFI-2) [76, 81]

### Participant timeline {13}

Participants will receive 20 days of therapy, 10 days with active current, and 10 days sham stimulation.

Before each block, primary, secondary, and other outcomes will be assessed. In addition, two follow-ups will be performed, with 2 days of assessments each. Finally, there will be a close-out, with complete evaluation of all outcomes (Fig. 4).

### Sample size {14}

Sample size calculations were conducted in G*Power 3.1.9 for the repeated measures, within-between interaction (ANOVA). The total sample size needed to detect a high assessment-by-treatment interaction effect (*f* = 0.25, beta = 0.95, alpha = .05, groups = 2, measurements = 5) is 32 participants. However, to compensate for a high estimated dropout rate of up to 25% during treatment, the aim is to recruit 40 participants leading to 20 participants per group.

### Recruitment {15}

The recruitment is taking place at the USZ, during evaluations by otorhinolaryngologists. Additionally, there is a website available with the study information, from which interested individuals can learn more about the study and register their interest in trial participation.

## Assignment of interventions: allocation

### Sequence generation {16a}

Specific prognostics may influence the outcomes; therefore, the intervention groups will be balanced to minimize this influence. Randomized group assignment will be performed using pair matching for the two study arms, by way of stratification according to age, sex, hearing loss, and tinnitus severity.

Despite already excluding individuals with slight and catastrophic THI, mean levels in grades 2 to 4 have different impacts among participants [87]. Thus, to obtain this control, participants will be allocated so that both groups will present individuals with the same levels of tinnitus severity.

Randomization will be performed by a software script optimizing group allocation along the defined parameters in a pair-matching manner. The entire study sample will be split in half for the two study arms and random 3-digit strings will be used for all participants.

### Concealment mechanism {16b}

Randomization is performed on pseudonymized study codes with no name or other personal identifiers (i.e., sex, age in years, and tinnitus severity with the THI score).

Randomization is performed by the biometrician who is a researcher independent of the actual study procedures. The allocation list is kept in a separate file on the independent computer, only the biometrician has access to the document.

### Implementation {16c}

The enrollment of participants is performed by two professionals who are not participating in laboratory procedures. The allocation sequence and the assignment of participants to interventions will always be performed by the biometrician who also is never part of any treatment visits or procedures.

## Assignment of interventions: Blinding

### Who will be blinded {17a}

Participants and researchers performing the treatments and measurements will be blinded. The information about the course of the training is kept identical for participants in both groups: the position of the electrodes is the same, and the duration of stimulations and sessions are the same for both groups. In addition, the current in the sham condition is applied during the first and last 30 s of stimulation as a ramp up and ramp down, respectively, thus the participant feels a tingling sensation similar to the active stimulation. TRNS and AS is programmed in such a way that the study personnel are blinded to the active or sham status of these interventions.

### Procedure for unblinding if needed {17b}

An Emergency Code Break will be available to the investigator. This Code Break should be opened only in emergency situations when the identity of the intervention must be known by the investigator in order to provide appropriate medical treatment.

## Data collection and management

### Plans for assessment and collection of outcomes {18a}

Given the study’s longitudinal design, including constant and periodic assessments, data will be constantly evaluated.

The assessors are the researchers of the study. They are trained to perform standardized care, the way of approaching the participant and carrying out the assessment and directions during treatment.

The questionnaires are answered without the presence of the evaluator and without a time limit, so that the answer is not influenced by external factors. Likewise, all questionnaires used in the study are validated and consolidated in the literature.

PM will be performed twice in order to increase the reliability of the critical tinnitus frequency matchings.

### Plans to promote participant retention and complete follow-up {18b}

No specific plans to promote participant retention are implemented in this trial. As this is a trial involving treatment for a symptom that so far has no cure, participant retention is not usually an issue in the treatment phase.

As the treatment is over an extended period of time, it is possible that sessions may be forgotten or forms not filled out and, in these cases, participants will be contacted by phone and email.

### Data management {19}

The data is organized in a pseudonymization allocation list. Each participant receives one code after the randomization, generated by a computer program, which will be used throughout the trial. The participants will be informed about this procedure before starting the data collection. Participant data may only be reviewed by authorized persons on the research team or other authorized people to verify that the study is being accomplished correctly. All persons authorized will have a duty of confidentiality.

All collected data are answered online on a secure platform, then stored in computer files protected by the IT department of University Hospital Zurich. The pseudonymization allocation list is kept separate from the pseudonymized data, by the biometrician, and deleted upon completion of the trial. Data are kept for 10 years before being deleted.

### Confidentiality {27}

The investigators will treat the entire body of information related to the study and the compiled data strictly confidentially. Any forwarding of information to persons not directly involved in the study must be approved by the owner of the information.

Data generation, transmission, archiving, and analysis of personal data within this study strictly follows the current Swiss legal requirements for data protection. A prerequisite is the voluntary approval of the participant given by signing the informed consent form prior to the start of participation in the clinical trial.

The participant medical information obtained for the study is confidential. Participant’s confidentiality will be further ensured by utilizing participant identification code numbers to correspond to treatment data in the computer files.

Such medical information may be given to the participant’s personal physician or to other appropriate medical personnel responsible for the participant’s welfare, if the participants have given their written consent to do so.

Data generated as a result of this study will be available for inspection on request by the monitors and by the Competent Ethics Committee.

### Plans for collection, laboratory evaluation, and storage of biological specimens for genetic or molecular analysis in this trial/future use {33}

This is not applicable, no samples collected.

## Statistical methods

### Statistical methods for primary and secondary outcomes {20a}

The distributions of the data will be assessed using measures of central tendency and measures of dispersion to determine the characteristics of tinnitus and describe other variables associated with the symptom.

The descriptive tinnitus data will be presented in tables, separated by intervention group. The safety parameters, adverse events, and laboratory assessments will be listed by participants and displayed in summary tables.

Finally, the assumptions of the independent and dependent variables will be evaluated. Parametric or non-parametric tests will be chosen based on the features of the data. These tests include the verification of absence of outliers through boxplot; homogeneity of variances in the independent variables through Levene test; and Mauchly’s test for sphericity, or if necessary, Greenhouse Geisser corrections.

The analysis will be performed using the statistic program R (https://www.r-project.org).

#### Primary analysis

The data will be statistically analyzed for changes in subjective tinnitus loudness and distress (VAS) and MML by means of frequentist linear mixed-effect modelling (LME) by the biometrician after data collection has been completed.

Between- and within-subjects repeated measures ANOVA will be used to compare the effect of treatment pre and post, in both intervention groups.

#### Secondary analysis

Regarding the EEG outcomes, pre-to-post induced changes in EEG resting-state patterns and ERP data will be analyzed. Analysis will be performed in MATLAB Software (Mathworks, Natick, MA) [46], in FieldTrip toolbox [88]. This encompasses preprocessing, power, and connectivity analyses.

### Interim analyses {21b}

No interim analyses are scheduled for this trial.

### Methods for additional analyses (e.g., subgroup analyses) {20b}

Each intervention group will also be compared separately using LME and respective post hoc tests when necessary. This will be to observe the effect of HD-tRNS alone and bimodal stimulation (HD-tRNS and AS associated).

### Methods in analysis to handle protocol non-adherence and any statistical methods to handle missing data {20c}

Efficacy analyses will be performed according to the intention-to-treat principle. The participants will be analyzed in the treatment arms to which they were randomized, irrespective of whether they refused or discontinued the treatment, or whether other protocol violations occurred. Since the questionnaires are collected electronically and each item is mandatory for questionnaire completion, we expect a low number of missing data. The other self-report assessments are also carried out electronically by standard, so that the number of missing data should also be low here. Should missing data nevertheless occur, this will be handled as follows:

The missing numerical data will be filled according to the average value of all participants or a more fitting procedure of data imputation depending on the specific issue (e.g., median or *k* nearest neighbor). Non-numerical missing data are listed as missing data accordingly.

### Plans to give access to the full protocol, participant-level data, and statistical code {31c}

The data, without identifying the participants, will be made available upon reasonable request.

## Oversight and monitoring

### Composition of the coordinating center and trial steering committee {5d}

The trial is coordinated and steered by the investigators of this study. They are responsible for the design and conduct of the study, for overseeing adherence to the study protocol, for the preparation of protocol and revisions, and the publication of study reports.

This study will be performed in collaboration with the University Hospital Zurich.

The hospital committee is responsible for the regulatory compliance, clinical monitoring, source data verification, and quality management.

### Composition of the data monitoring committee, its role and reporting structure {21a}

The cantonal ethics committee approved the conduction of this study after reviewing the project protocol and participant’s risk. According to national guidelines, data monitoring can be performed by the investigators themselves. Changes to the study protocol are reported to the ethics committee. In addition, the ethics committee is informed annually about the status of the study. In the event of a serious adverse event, the ethics committee will be informed immediately. The committee is independent and has no conflict of interest with this study.

### Adverse event reporting and harms {22}

Neuromodulation is a safe procedure, causing no apparent short- or long-term harm.

Only low adverse events are possible, such as headaches, burning, itching, tingling, tiredness, heat, redness, irritation, difficulty concentrating, and pain. However, the side effects will be monitored after each session through a safety questionnaire. In cases of the appearance of secondary effects to the treatment, the investigators are requested to report any untoward clinical event on the adverse event page of the case report form (CRF). All details will be reported, including the time of occurrence, symptoms and signs, severity, duration, and causal relationship with the treatment.

In case of discomfort to adverse events, the case will be analyzed by the health professionals involved, who will decide whether changes in the treatment could benefit the participants and reduce the side effects or whether participation in the study should be terminated.

### Frequency and plans for auditing trial conduct {23}

At the University Hospital Zurich, there will be irregular audits in order to determine whether appropriate processes are in place, procedures are adequate, the researcher is appropriately qualified to take on their tasks, and important documents such as case report forms and protocol are attached.

### Plans for communicating important protocol amendments to relevant parties (e.g., trial participants, ethical committees) {25}

Any modifications to the trial protocol are communicated and approved by the ethics committee. After approval of the ethics committee, the documents are introduced into practice.

In case of important protocol modifications which may have an impact on the conduct of the study, these will also be communicated to the trial registries.

### Dissemination plans {31a}

Upon trial completion, the results will be reported and disseminated in an international peer-reviewed journal and presented at academic conferences, irrespective of the results of the trial.

No restrictions are imposed from either investigators or other interested parties.

## Discussion

The main objective of this study is to explore the added clinical value of bimodal (electric and acoustic) stimulation in chronic tinnitus patients. In order to ascertain valid results, this approach is compared to unimodal electric stimulation and sham conditions. Besides that, we aim at identifying what differentiates groups of responders and non-responders to provide potential clinical guidelines and classify subgroups of tinnitus patients based on several objective and subjective measurements. Tinnitus is the result of functional changes in neuronal activity, which justifies the use of neuromodulation for the treatment of the symptom [13]. Among the neuromodulation types, tRNS has been proven to show the most positive treatment effect for tinnitus-related outcomes [24, 35]. Recent studies indicate that lf-tRNS might be superior to hf-tRNS and wf-tRNS [53], even if the optimal frequency range is still under debate. Bimodal stimulation has been evaluated to be more effective in neuroplastic effects than isolated treatments and, e.g., lead to a clinically relevant reduction in THI scores [89]. Furthermore, the treatment effect could be sustained for 12 months after treatment [89]. To the best of our knowledge, there has been no study which evaluated the influence of HD-tRNS applied over the auditory cortex bilaterally when combined with AS for the reduction of tinnitus loudness and distress.

In addition, a central novelty of the study is the use of a HD-tRNS allowing greater precision of the target location through the use of 17 HD electrodes. The wide range of audiometric and tinnitometric measurements will allow us to build subgroups based on hearing exams as well as other outcome measures and enable more reliable and valid results. Reactivity to residual inhibition for example could give insights on treatment effects of the acoustic stimulation and help to determine best clinical practices. Another theoretical advance may be considered in the assessment of individual tinnitus frequency through pitch matching which then forms the basis of the ERP paradigm. With a neural response on individually adjusted pure tones, further neuroplastic changes in the auditory cortex after the HD-tRNS can be explored. The paradigm is an iteration of a recent study by [53]. In more detail, we expect a normalization of ERP responses in tinnitus sufferers [90]. Ideally, collected data will therefore contribute to the discussion of pathological auditory perception and finally may act as a biomarkers or classifiers of chronic tinnitus patients [51].

With the double-blinded crossover design, it will be possible to evaluate the efficacy of HD-tRNS and bimodal stimulation compared to sham conditions. The between-subject comparison will allow us to investigate the added value of combined stimulation and the unspecific effect of any acoustic input during tES. The use of double-blind design enables a proper evaluation of the treatment approaches by reducing biasing influences of the study personnel. Unfortunately, the design does not include a branch with acoustic stimulation only and a control group of healthy hearing matched participants is also missing. These shortcomings are due to the focus of the study as well as resource and infrastructure limitations. It was thus decided to focus on the effectiveness of electric stimulation and its combination with acoustic stimulation. Supplementary long-term follow-up measurements could additionally contribute further efficacy data of bimodal stimulation in reducing tinnitus loudness and distress.

Among the limitations, it might be necessary to consider the individual anatomical brain characteristics of each participant and different brain areas as potential targets [4–7] instead of using a generalized approach based on the localization of Rademacher et al. [43].

It is also necessary to consider the variability of the tinnitus characteristics among the participants to determine optimal parameters in terms of rate, level, mode, and pattern of stimulation [91]; thus, in order to obtain a more homogeneous sample, specific exclusion criteria were included, such as hearing loss and tinnitus severity.

Multiple sessions of tRNS show an increase in the suppression effect when compared with single session [35], but there are not enough studies that verify the ideal number of sessions to detect change in tinnitus perception. However, Shekhawat and Vanneste showed stabilization of the effect after the sixth session [92] which confirms our choice of 10 active stimulation sessions in rapid succession, even if the ideal treatment time and the length of any lasting effects are still being investigated.

Despite the fact that there are treatment options for tinnitus such as cognitive behavioral therapy [93], it is widely acknowledged that an established treatment to reduce tinnitus loudness does not yet exist [9, 13]. Thus, the purpose of the present study is to identify a treatment method, which provides long-term changes in tinnitus loudness and thus improve the quality of life in a population of chronic tinnitus patients. As the wide heterogeneity influences the variability in treatment response [94], our data will furthermore contribute to the discussion of objectively subtyping groups of tinnitus sufferers and finding personalized treatment methods.

Taken together, this study includes different technical novelties such as focal targeting of Heschl’s gyrus bilaterally through HD-tRNS or the individualized auditory ERP paradigm. With the broad spectrum of objective measurements, we seek to establish a dataset as proposed by the mechanistic-driven precision medicine framework [95]. Bimodal stimulation is a promising neuromodulation method in improving tinnitus symptoms [13]. Finally, given the causal implications of electric brain stimulation, it may further the understanding of the neural mechanisms underlying tinnitus.

## Trial status

Ethics Committee Protocol Version 3.2 of April 14, 2021.

Recruitment starts in April 2021 and is expected to be completed in December 2022.

## Data Availability

The data will be made available upon reasonable request.

## Abbreviations

AS: Acoustic stimulation
BAI: Beck-Angst-Inventar
BDI: Beck-Depressions-Inventar
BFI-2: Big Five Inventory
CRF: Case report form
DLPFC: Dorsolateral prefrontal cortex
EEG: Electroencephalogram
GÜF: Geräusch-Überempfindlichkeits-Fragebogen
HD-tRNS: High-definition transcranial random noise stimulation
hf: High frequency
IFMC: Isometric forward masking contour
LDL: Loudness discomfort level
lf: Low frequency
LME: Linear mixed-effect modelling
MD: Medical device
MML: Minimum masking level
PM: Pitch matching
PTA: Pure tone audiometry
RI: Residual inhibition
rTMS: Repetitive transcranial magnetic stimulation
SL: Sensation level
tACS: Transcranial Alternating Current Stimulation
tDCS: Transcranial direct current stimulation
tES: Transcranial electric stimulation
TFI: Tinnitus Functional Index
THI: Tinnitus Handicap Inventory
TMC: Temporal masking curve
tRNS: Transcranial random noise stimulation
TSCHQ: Tinnitus Sample Case History Questionnaire
USZ: University Hospital Zurich
UZH: University of Zurich
VAS: Visual analog scale
wf: Whole frequency
WHOQoL: World Health Organization Quality of Life
WN: White noise
